# Deterioration in Muscle Mass and Physical Function Differs According to Weight Loss History in Cancer Cachexia

**DOI:** 10.3390/cancers11121925

**Published:** 2019-12-03

**Authors:** Guro Birgitte Stene, Trude Rakel Balstad, Anne Silja M. Leer, Asta Bye, Stein Kaasa, Marie Fallon, Barry Laird, Matthew Maddocks, Tora S. Solheim

**Affiliations:** 1 Department of Neuromedicine and Movement Science, Faculty of Medicine and Health Sciences, NTNU—Norwegian University of Science and Technology, 7491 Trondheim, Norway; annesiljaleer@gmail.com; 2 Department of Clinical and Molecular Medicine, Faculty of Medicine and Health Sciences, NTNU—Norwegian University of Science and Technology, 7491 Trondheim, Norway; trude.r.balstad@ntnu.no (T.R.B.); stein.kaasa@ntnu.no (S.K.); barry.laird@ed.ac.uk (B.L.); tora.s.solheim@ntnu.no (T.S.S.); 3 Cancer Clinic, Trondheim University Hospital, 7491 Trondheim, Norway; 4 Department of Nursing and Health Promotion, Faculty of Health Sciences, OsloMet—Oslo Metropolitan University, 0167 Oslo, Norway; abye@oslomet.no; 5 Regional Advisory Unit for Palliative Care, Department of Oncology, Oslo University Hospital, 0167 Oslo, Norway; 6 European Palliative Care Research Centre (PRC), Department of Oncology, Oslo University Hospital and Institute of Clinical Medicine, University of Oslo, 0167 Oslo, Norway; 7 Edinburgh Cancer Research Centre, University of Edinburgh, Crewe Road, Edinburgh EH4 2XR, UK; Marie.Fallon@ed.ac.uk; 8 Institute of Genetics and Molecular Medicine, University of Edinburgh, Edinburgh EH4 2XR, UK; 9 St Columba’s Hospice, Boswall Road, Edinburgh EH4 2XR, UK; 10 Cicely Saunders Institute of Palliative Care, Policy & Rehabilitation, King’s College London, Bessemer Road, London SE5 9PJ, UK; matthew.maddocks@kcl.ac.uk

**Keywords:** cachexia, weight loss, muscle mass, grip strength, physical performance, cancer, endpoints

## Abstract

Background: Muscle mass and physical function (PF) are common co-primary endpoints in cancer cachexia trials, but there is a lack of data on how these outcomes interact over time. The aim of this secondary analysis of data from a trial investigating multimodal intervention for cancer cachexia (ClinicalTrials.gov: NCT01419145) is to explore whether changes in muscle mass and PF are associated with weight loss and cachexia status at baseline. Methods: Secondary analysis was conducted using data from a phase II randomized controlled trial including 46 patients with stage III–IV non-small cell lung cancer (n = 26) or inoperable pancreatic cancer (n = 20) due to commence chemotherapy. Cachexia status at baseline was classified according to international consensus. Muscle mass (assessed using computed tomography (CT)) and PF outcomes, i.e., Karnofsky performance status (KPS), self-reported PF (self-PF), handgrip strength (HGS), 6-minute walk test (6MWT), and physical activity (PA), were measured at baseline and after six weeks. Results: When compared according to cachexia status at baseline, patients with no/pre-cachexia had a mean loss of muscle mass (−5.3 cm^2^, *p* = 0.020) but no statistically significant change in PF outcomes. Patients with cachexia also lost muscle mass but to a lesser extent (−2.8 cm^2^, *p* = 0.146), but demonstrated a statistically significant decline in PF; KPS (−3.8 points, *p* = 0.030), self-PF (−8.8 points, *p* = 0.027), and HGS (−2.7 kg, *p* = 0.026). Conclusions: Weight loss history and cachexia status at baseline are of importance if one aims to detect changes in PF outcomes in cancer cachexia trials. To improve the use of co-primary endpoints that include PF in future trials, outcomes that have the potential to detect change relative to weight loss should be investigated further.

## 1. Introduction

Cancer cachexia is a highly prevalent metabolic wasting syndrome characterized by involuntary weight loss, accompanied with a progressive loss of skeletal muscle mass as a key diagnostic criterion [1]. The condition cannot be fully reversed by conventional nutritional support and leads to progressive functional impairment, reduced quality of life and increased mortality [2]. There are no current licensed drug treatments for cancer cachexia and the severity of the condition warrants an urgent need to find treatment alternatives [3].

Consequently, multiple clinical trials aim to attenuate this condition [4,5,6,7]. Commonly, these trials measure the effects of interventions using outcomes that capture changes in key cachexia domains, including body weight or muscle mass and physical function (PF) [8]. It has been shown that low muscle mass is associated with reduced PF [9,10] and by definition, cancer cachexia should also lead to a deterioration of PF [1]. Thus, sound methods for assessing various dimensions of PF are justified to measure the effects of anticachexia treatments [1]. Consequently, regulatory agencies have also put forward the need for outcomes that capture both changes in muscle mass and PF as co-primary endpoints in clinical trials [11].

Recent trials, such as the ROMANA trials investigating the effects of Anamorelin, have used change in lean body mass (LBM) and PF measured as handgrip strength as co-primary endpoints [4]. These trials demonstrated significant increases in lean body mass (LBM) in favor of Anamorelin but observed no corresponding changes in PF outcomes. In the POWER 1 and 2 trials, preliminary data presented favorable effects of Enobosarm on LBM in both trials, but for the co-primary endpoint, corresponding effect was found only in the POWER 1 trial (≥10% increase in stair climb power) [5,12]. Others have demonstrated large effect sizes on muscle mass (6.5 kg increase in LBM) in favor of a parental nutrition intervention, but still found no concurrent effect on PF outcomes [13]. Therefore, despite the success of these trials to impact on muscle mass, their inconsistency in inability to influence PF outcomes has led to failure of regulatory approval [14].

Recently, a model was developed to explain the non-linear relationship between changes in muscle mass and PF outcomes in cancer cachexia [15]. This model suggests that early in the cachexia pathway, PF can be maintained despite significant loss of muscle mass, thus measurable effects on PF outcomes are small. However, further on in the cachexia pathway when more muscle mass has been lost, PF declines at an increased rate, thus a measurable effect is more likely. This preposition is supported by data from Bye et al., who found that functional decline was most evident in patients with low muscle mass (skeletal muscle index below 42–45 cm^2^/m^2^ for men and 37–40 cm^2^/m^2^ for women) [9].

One hypothesis is that along the cachexia pathway, there is a variable impact on PF outcomes depending on the magnitude of changes in muscle mass, however, there is limited prospective data to support this idea. The few studies investigating this show inconsistent results [10,16]. If cachexia status and/or stage at baseline are of importance, this could help understand the inconsistency of PF outcomes, including the response to anticachexia intervention. As of today, these considerations have not been included as part of the design of cachexia intervention. In the ROMANA trials, patients were included if cachectic at baseline according to the cancer cachexia definition (involuntary weight loss of >5% within previous 6 months or BMI < 20 kg/m^2^) [4]. In the POWER trials, no minimum or maximum weight loss at baseline were required for eligibility [5].

We previously reported from a phase II randomized controlled trial, the pre-MENAC trial (ClinicalTrials.gov: NCT01419145) [17]. In this trial, investigating the feasibility of a multimodal intervention to treat cancer cachexia, it was demonstrated that a 6-week multimodal intervention (physical exercise, nutritional support and anti-inflammatory medication) is feasible and safe, but the analysis did not show a statistically significant effect on muscle mass and PF. The trial aimed to include patients that had a high risk of developing cachexia, i.e., patients who were in a pre-cachexia stage. Using data from this trial, we performed a secondary analysis aimed to explore whether cachexia status at baseline (no/pre-cachexia vs. cachexia) relates to the measurable effects on PF outcomes in cancer cachexia.

## 2. Results

### 2.1. Baseline Characteristics

Baseline characteristics for all patients and the subgroups (no/pre-cachexia and cachexia) are provided in Table 1. This study included 46 patients diagnosed with NSCLC stage III–IV (n = 26) and inoperable pancreatic cancer (n = 20). The mean score on the Karnofsky Performance Scale (KPS) was 87, corresponding to being able to do normal activity with effort and some signs or symptoms of disease [18]. According to the cancer cachexia definition [1], 16 patients were classified as having no/pre-cachexia, leaving 30 patients classified as having cachexia.

In the cachexia group, there were significantly more males (20/30) compared to the no/pre-cachexia group (6/16) and those patients who were cachectic had, as anticipated, significantly higher level of inflammation (measured by c-reactive protein (CRP)). There were no significant between-group differences with respect to age, body mass index (BMI), diagnosis and reported co-morbidities. In both groups, a little more than one-half of the patients were randomized to multimodal intervention.

### 2.2. Changes from Baseline to Week 6 (End of Trial) in Muscle Mass and PF Outcomes

Data on mean changes in muscle mass and PF outcomes are shown in Table 2. A significant decrease in muscle mass, skeletal muscle index (SMI), was found in the cachexia but not the no/pre-cachexia group. The no/pre-cachexia group had a significant decrease in KPS, self-reported PF and HGS, while there were no significant changes in PF outcomes for the cachexia group. Changes from baseline to week 6 were significantly different between the two groups for HGS only. Adjusting for trial arm allocation by ANCOVA did not alter the results.

### 2.3. Association between Changes in Muscle Mass and PF Outcomes from Baseline to Week 6 (End of Trial)

**Figure 1:**
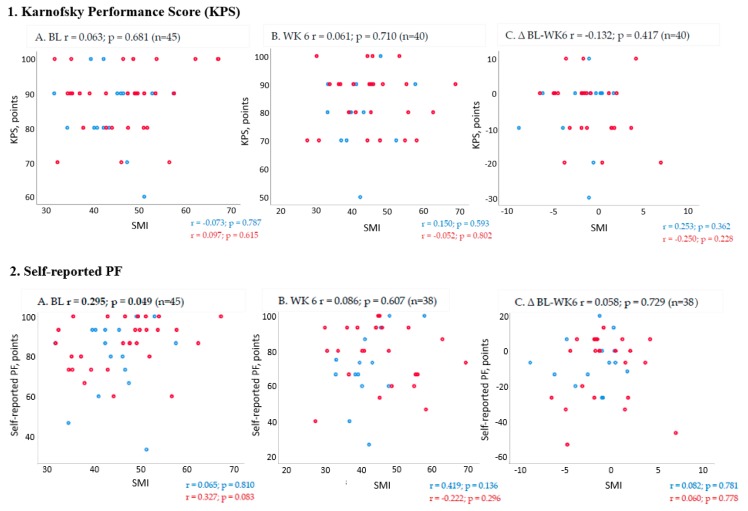

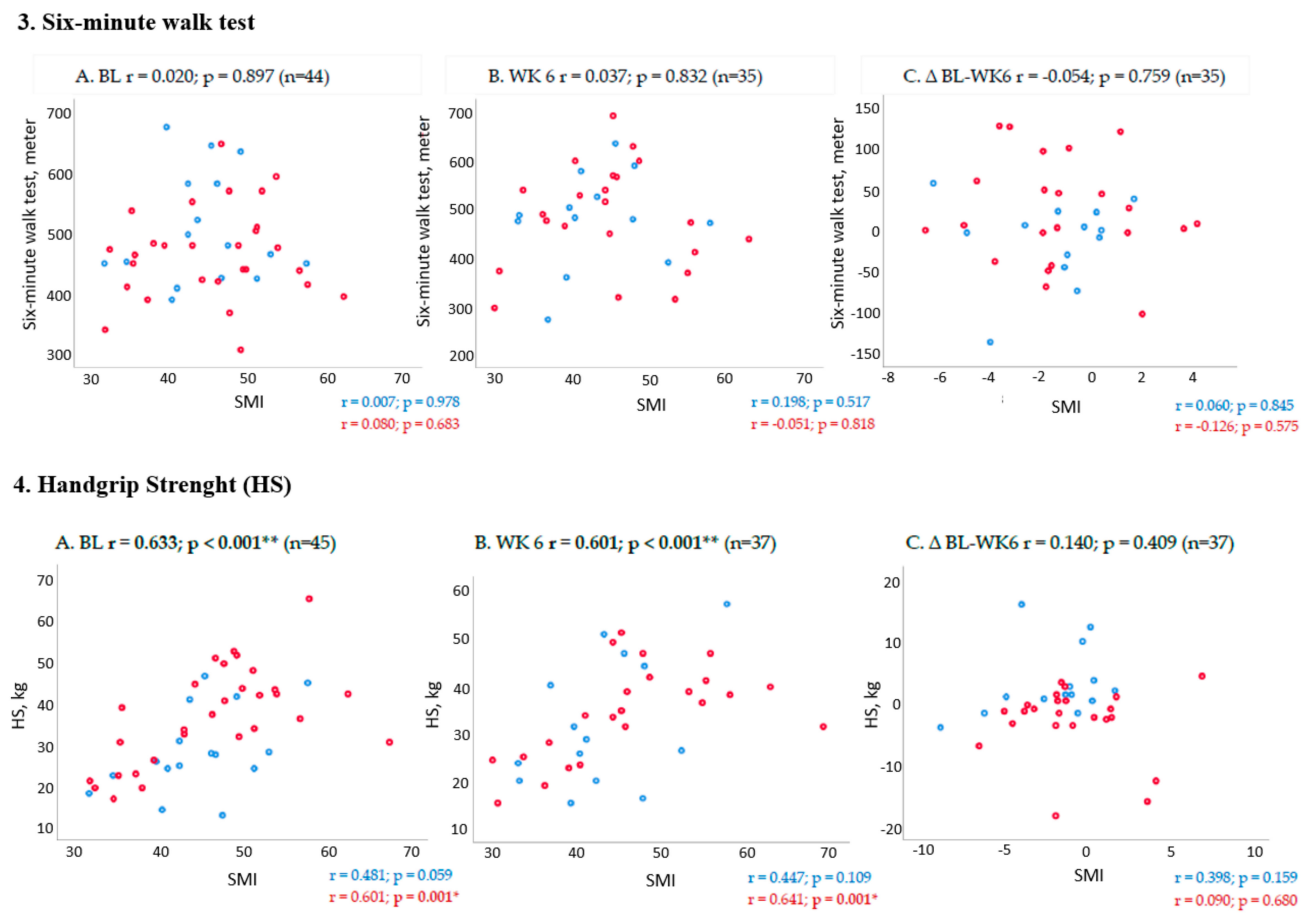

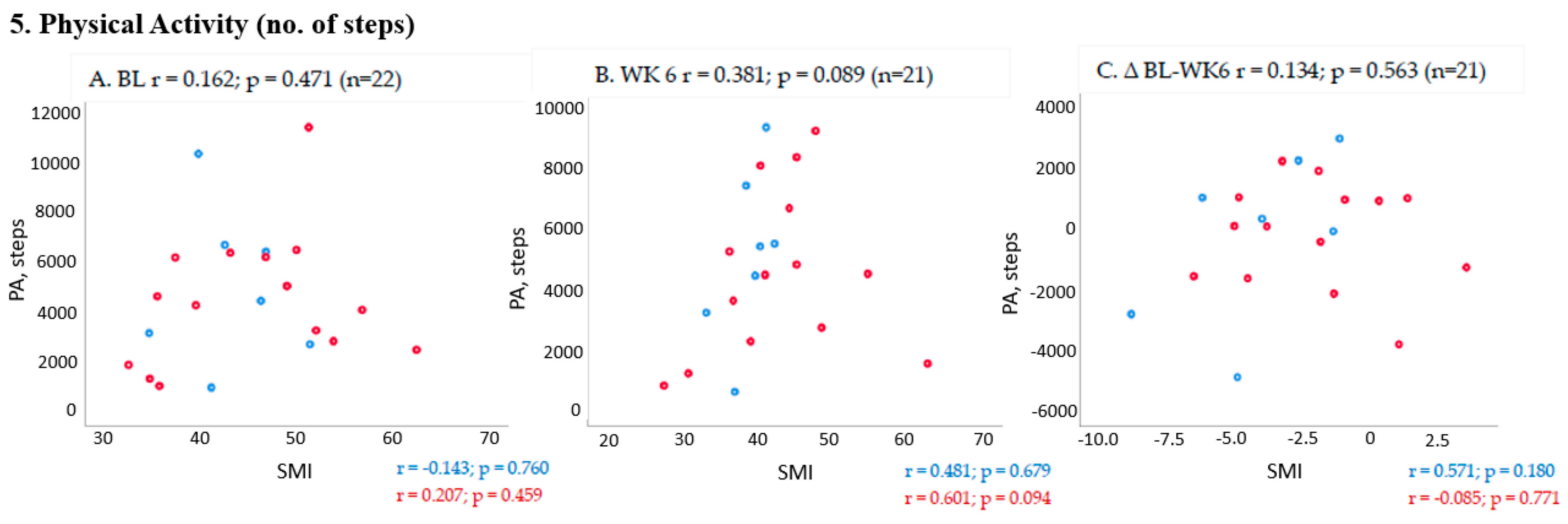
Scatterplots and Spearman’s Rho correlation coefficients (r) and p-values for skeletal muscle index (SMI) on the X- axis and the following physical function (PF) outcomes on the Y- axis 1. Karnofsky performance scale, 2. Self-reported PF, 3. Six-minute walk test, 4. Handgrip strenght and 5. PA, steps. Correlations are shown for all patients at (**A**) baseline (BL), (**B**) 6 weeks (WK 6) and (**C**) change from baseline to end of trial (∆ BL-WK 6). Statistically significant correlations are highlighted by bold types and the pre-fix * = correlation is significant at the 0.05 level, ** = correlation is significant at the 0.01 level. Sub-group analysis based on cachexia status at baseline are marked by blue dots=no/pre cachexia and red dots=cachexia in the scatter plots and correlation coefficients (r) and p-values are inserted below the plots for each sub-group.

Correlation coefficients (Spearman’s rho) for the association between muscle mass and PF outcomes is illustrated in Figure 1. For measurements of change from baseline to six weeks (end of trial) in all patients, there were no significant correlations between SMI and PF outcomes.

## 3. Discussion

In this study, we observed significant deterioration in PF after six weeks in patients who were defined as cachectic at baseline. These cachectic patients entered the trial at a stage where they already had lost a significant amount of body weight, including muscle mass. Further weight loss led to additional loss of muscle mass that eventually declined enough to impact on functionality. Furthermore, we observed that patients who were in early stage of cachexia (no/pre-cachexia) at baseline had a significant decline in muscle mass during the trial, yet without corresponding changes in PF outcomes. Our findings indicate that weight loss history is of importance in relation to understanding effects on PF outcomes in cancer cachexia.

Our findings thus support the hypothetical model proposed by Ramage et al. [15] suggesting that along the trajectory of cancer cachexia, there might be a variable impact on PF outcomes depending on the magnitude of weight loss. It is possible that patients that are included in clinical trials at an early stage in the development of cancer cachexia (no/pre-cachexia), will lose muscle mass but still be able maintain functionality during the trial as they have not “reached a threshold” in which muscle loss evokes measurable functional decline. Bye et al. previously identified such a threshold where SMI is low enough to evoke functional decline in a sample of advanced cancer patients [9]. This study was cross-sectional, so it can only suggest that patients must be beyond the no/pre-cachexia stage for functional decline to be observable. This is in line with findings from a prospective study where it was demonstrated that in patients who were cachectic at baseline, muscle depletion was accompanied by PF decline [10]. Still, others have identified subgroups within patients who are cachectic at baseline, who remain relatively stable over time with regards to changes in PF outcomes [16]. If there is indeed a variable impact on PF depending on the cachexia status of the patients, there is reason to debate whether a co-primary outcome, such as recommended by regulatory authorities, including both muscle mass and PF is feasible, especially considering that cachexia trials aim to include patients in the early phases of the cachexia trajectory.

However, an important aspect to discuss is the type of PF outcome opted for in cachexia trials as only modest correlations with muscle mass are so far demonstrated. Ideally, outcomes should be based on feasible measurement methods that are sensitive enough to capture subtle changes over time, and furthermore, in order to have clinical significance, these outcomes need to reflect everyday functioning in the cancer cachexia population [19]. PF outcomes of relevance in cachexia trials include methods that capture the patient’s own perception of their PF (e.g., self-reported PF from HRQoL questionnaires), or the health providers’ perception (e.g., observer-based indexes such as the Karnofsky performance status), or testing what the patients can do (e.g., physical performance testing such as the six-minute walk), or what the patient is actually doing in everyday life (e.g., free-living physical activity) [20].

A strength of the present study is that we have measurements of all these PF outcomes. As hypothesized, PF was significantly reduced in the groups that were cachectic at baseline. However, this was only the case for the outcomes of self-report PF, KPS, and HGS. In fact, the changes in 6MWT were small in this study and this did not change according to baseline cachexia status. More interestingly, change in handgrip strength were significantly different between the no/pre-cachexia and the cachexia group. The cachexia group had a significant decline of 2.7 kg from baseline to end of trial. The abundance of corresponding changes of muscle mass and grip strength reported in previous cancer cachexia trials could be due to that most patients in these studies had a baseline history of weight loss. Still, other explanations such as gender differences, the appropriateness of using upper limb strength versus leg strength, and the variability of measurement methods could also be relevant [15,21].

There are some limitations to the study. Based on a secondary analysis of data from a cancer cachexia trial, the sample size is small. There are some missing data on PF outcomes, mostly for patients classified as cachectic at baseline and for the grip strength measurements; missing data were from women mostly. Statistical analysis adjusting for gender or inflammation was not the scope of this study and was not performed due to sample size considerations. However, the observed differences in changes in handgrip strength between patients with no/pre-cachexia and cachexia must be considered bearing in mind gender differences in the compared groups [22]. We did not perform analysis to adjust for the intervention/control arm in this study, as sample size was small and there were almost equal numbers of patients receiving multimodal treatment in the no/pre-cachexia and cachexia groups. Still, the effect of the intervention relative to weight loss history and change scores in muscle mass and PF outcomes, remains to be investigated. It is possible that changes in muscle mass in patients with advanced cancer can be explained by factors such as fluid retention, tumor growth, or inflammation. To quantify muscle mass, we made calculations from CT-scans, which is a gold standard technique that separates skeletal muscle from other tissue volumes [23,24].

We therefore argue that it is necessary to further explore longitudinal data from observational studies that focus both on cachexia characteristics and psychometric properties of relevant outcomes of functionality to guide choices of relevant endpoints. As the relationship between muscle mass and PF outcomes is still unclear, further studies to estimate the magnitude of muscle mass increase needed to evoke an increase in PF are necessary to determine the clinical benefit of interventions aimed at attenuating weight loss in advanced cancer. Ideally, different functional trajectories within the cancer cachexia population should be identified and characterized to inform future clinical trials.

## 4. Materials and Methods

### 4.1. Study Design and Participants

This study is based on data from 46 patients included in a phase II randomized controlled trial conducted between 2010 and 2014 investigating the feasibility of a multimodal intervention to treat cachexia in patients with advanced cancer—the pre-MENAC study (ClinicalTrials.gov: NCT01419145) [17]. Patients diagnosed with stage III–IV non-small cell lung cancer and patients with inoperable pancreatic cancer, KPS ≥ 70, BMI < 30 kg/m^2^, and <20% weight loss in the previous six months were eligible. The eligibility criterions aimed to include patients that had newly developed cachexia or had a high risk of developing the condition. Patients were randomly allocated into two arms, either multimodal treatment (exercise, omega-3 enriched oral nutritional supplement, and Celecoxib) or control (standard care). Two cycles of standard chemotherapy were scheduled during the trial period. The pre-MENAC study protocol was approved by ethics committees for human research (Study ID number 2010/2620) and medical agencies at the participating centers (EudraCT number 2010-022897-14) and written informed consent was obtained from all participants.

### 4.2. Data Collection Procedures

Data used in the current study were collected at baseline and at the study endpoint following a six-week intervention period. Baseline measurements included demographics (age and gender), tumor type and stage, comorbidities, measured height, body weight in kilograms, and self-reported weight loss during the last 6 months. Body mass index (BMI) was calculated from body weight and height (kg/m^2^). Data collected both at baseline and at week six were muscle mass and the following PF outcomes: Karnofsky performance status, handgrip strength, six-minute walk, free-living physical activity (No. of steps) and self-reported PF.

### 4.3. Measures of Muscle Mass

Muscle mass was measured from computerized tomography (CT) images of the thorax/upper abdomen taken as part of study protocol. Images had to be taken within four weeks of baseline measurements and within a period of one week prior to or one week following study endpoint (week six). Cross-sectional imaging at the third vertebral level (L3) is the preferred method for quantification of muscle mass as it is highly correlated to total body muscle mass [25]. Slice-O-Matic© software (v.4.3, Tomovision, Montreal, QC, Canada) was used to quantify the cross-sectional area (CSA, cm^2^) of the muscles at L3 within a Hounsfield Unit range of −29 to +150. A skeletal muscle index (SMI) was calculated from the cross-sectional area (cm^2^) divided by squared height (m^2^) and expressed as SMI (cm^2^/m^2^). The SMI was used as a variable in the statistical analysis.

### 4.4. Measures of Physical Function

Karnofsky performance scale (KPS) is an observer-based and clinically orientated measure that is commonly used as entry criteria into oncology trials [18]. KPS classifies the functional impairment of patients on a scale from 0 (death) to 100 (normal function), with intervals of 10, with higher scores indicating better function [26]. Self-reported PF (self-PF) was obtained from the European Organization for Research and Treatment of Cancer Quality of Life Questionnaire C30 (EORTC QLQ-C30) [27]. The PF domain includes five items that query about the self-perceived degree of difficulty in performing activities of daily living, ranging from more strenuous activities such as taking a long walk to self-care activates such as eating, dressing, going to the toilet, etc. All items are scored on a 4-point ordinal scale. The numbered scale represents “not at all” = 1, “a little” = 2, “quite a bit” = 3 and “very much” = 4. Before statistical analyses, the scale scores were linearly transformed to a 0–100 scale as recommended in the EORTC scoring manual [28]. Higher scores on the scale represent better functioning. Differences of 10 points or more are usually regarded as clinically significant [29]. Grip strength (HGS) was measured with a hydraulic hand-held dynamometer (JAMAR). The test was performed using the dominant hand and three test trials were performed. In the analysis, the mean of the three test trials was used as outcome. The 6MWT was performed in a 30-meter hallway where the patients walked back and forth between two points marked by cones [30]. Total number of meters walked was recorded at the completion of the test and used as outcome. Free living physical activity (PA) was assessed by the ActivPAL^®^ which is a body worn electronic sensor that is worn on the thigh of the patients [31]. Mean number of steps taken (step count) was used as outcome in this study as this is validated in cancer cachexia [19].

### 4.5. Data Analysis and Statistics

As the aim of the present study was not to evaluate the effect of the intervention, the primary analysis was performed on data pooled from both arms from the trial. Additional analysis was performed to adjust for arm allocation. To account for cachexia status at baseline, the pooled sample were classified according to the international consensus definition of cancer cachexia as proposed by Fearon et al. [1]. Cachexia is defined as >5% weight loss during the last six months or weight loss more than 2% and BMI < 20 or sarcopenia (defined as SMI for males <55 cm^2^/m^2^); for women <39 cm^2^/m^2^) and any weight loss. Patients not classified as having cachexia according to this definition were defined as no cachexia/pre-cachexia. Descriptive statistics are presented by mean values and standard deviation for continuous variables and frequencies for proportions (number/%) for categorical data. To test for differences in baseline characteristic between groups classified according to the cancer cachexia definition (no/pre-cachexia and cachexia), parametric (two sample *t*-tests) and non-parametric tests (Mann–Whitney) were used. Primary analysis of change from baseline to week six (end of trial) was calculated by taking the measurement at week six minus the baseline measurement for muscle mass and PF outcomes. Paired and independent sample *t*-tests were used to compare change within and between groups in muscle mass and PF outcomes. Univariate analysis of co-variance (ANCOVA) was performed to adjust for the arm allocation. Statistical analyses were performed using IBM Statistics 25 software. The level of statistical significance was set at 5%.

## 5. Conclusions

This study highlights the complex relationship between changes in muscle mass and PF outcomes that are commonly used in cancer cachexia trials. Interpretation of the results indicate that the baseline cachexia status of patients entering a cancer cachexia trial is of importance if aiming to detect changes in PF and that this potentially explains the lack of effects in co-primary endpoints used in previous cancer cachexia trials. We argue that the optimal PF outcomes are yet to be defined in the cancer cachexia setting and that for such outcomes to be acknowledged, their potential to detect change relative to weight loss should be subject to further investigation.

## Figures and Tables

**Table 1:** Baseline characteristics for all patients and patients defined by cachexia status at baseline.

Variables/Statistics	All Patients (n = 46)	No/Pre-Cachexia (n = 16)	* Cachexia (n = 30)
Age, mean (SD)	59.8 (8.4)	60.4 (9.3)	59.5 (8.3)
Gender, male, n (%)	26 (57)	6 (38)	20 (67)
Diagnosis, n (%)			
NSCLC; Stage III	5 (11)	2 (12.5)	3 (10)
NSCLC, Stage VI	21 (46)	9 (56)	12 (40)
Pancreas, Stage I–III	11(24)	3 (19)	8 (27)
Pancreas, Stage IV	9 (19)	2 (12.5)	7 (23)
Weight loss, %, mean	6.1 (7.1)	0.3 (6.5)	9.3 (5.1)
C-reactive protein, mg/L, mean	25.7 (32.3)	11.8 (16.9)	32,5 (35.9)
BMI, kg/m^2^, mean (SD)	23.8 (3.6)	24.6 (3.5)	23,3 (3.6)
SMI, cm^2^/h^2^, mean			
Women	39.0 (5.4)	41.5 (5.7)	36.2 (3.5)
Men	50.3 (6.5)	49.3 (5.2)	50.6 (6.9)
Grip strength, kg, mean			
Women	24.3 (5.8)	23.8 (4.8)	24.8 (6.9)
Men	40.7 (10.1)	35.6 (13.0)	42.2 (8.9)
KPS, mean (SD)	87.2 (9.6)	85 (10.3)	88.3 (9.1)
self-PF score, mean	84.2 (15,2)	79.6 (19.2)	86.7 (12.3)
6-min walk test, meter, mean	481.6 (83.0)	505.5 (90.9)	468.4 (76.7)
PA, No. of steps, mean	4549.9 (2759.8)	4865.9 (3141.2)	4402.5 (2668.9)
Co-morbidities, n (%)			
Cerebrovascular disease	1 (2)	0	1 (3)
Chronic pulmonary disease	5 (11)	3 (19)	2 (7)
Liver disease	1 (2)	1 (6)	0
Diabetes mellitus	5 (11)	0	5 (17)
Other	18 (39)	7 (44)	11 (37)
None	14 (30)	5 (31)	9 (30)
Randomized to multimodal treatment, n (%)	25 (54)	9 (56)	16 (54)

BMI = body mass index; KPS = Karnofsky performance status; NSCLC = non-small cell lung cancer, PF = physical function, PA = physical activity; * Cachexia is defined as weight loss (last six months) >5% or weight loss >2% and BMI < 20 or sarcopenia (defined as skeletal muscle index for males < 55 cm^2^/m^2^); women < 39 cm^2^/m^2^) + any weight loss. Patients not classified as having cachexia according to this definition, was classified as no cachexia/pre-cachexia.

**Table 2:** Change scores (mean and SD) from baseline to week six for muscle mass and PF outcomes in all patients and groups based on cachexia status at baseline.

		All Patients	No/Pre-Cachexia	Cachexia
SMI, cm^2^/m^2^	n	40	15	25
	Baseline	45.7 (8.6)	44.6 (6.8)	46.4 (9.6)
	6 weeks	44.4 (9.1)	42.6 (6.8)	45.4 (10.3)
Within group	∆	−1.3 (3.1) CI: 0.3 to 2.3	−2.0 (2.9) CI: 0.4 to 3.6	−0.9 (3.2) CI: −0.4 to 2.2
	*p*-value	0.010 *	0.019 *	0.155
Between group	∆		−1.0 (1.0) CI: −3.1 to 1.0	
	*p*-value		0.308	
KPS, score	n	41	13	26
	Baseline	87.1 (9.8)	84.0 (9.9)	88.9 (9.5)
	6 weeks	83.2 (11.3)	80.0 (12.5)	85.0 (10.3)
Within group	∆	−3.9 (8.9) CI: 1.1 to 6.7	−4.0 (9.9) CI: −1.5 to 9.5	−3.8 (8.5) CI: 0.4 to 7.3
	*p*-value	0.008 **	0.138	0.030 *
Between group	∆		−0.2 (2.9) CI: −6.1 to 5.8	
	*p*-value		0.958	
Self-PF, points	n	39	14	25
	Baseline	83.8 (15.1)	77.1 (19.3)	87.5 (10.9)
	6 weeks	75.8 (18.7)	70.6 (20.9)	78.7 (17.1)
Within group	∆	−8.0 (17.0) CI: 2.5 to 13.5	−6.5 (13.8) CI: −1.4 to 14.5	−8.8 (18.7) CI: 1.1 to 16.5
	*p*-value	0.006 **	0.099	0.027 *
Between group	∆		2.3 (5.7) CI: −9.4 to 13.9	
	*p*-value		0.696	
6MWT, meters	n	36	13	23
	Baseline	472.3 (81.9)	492.7 (81.9)	460.8 (81.5)
	6 weeks	482.0 (101.1)	481.4 (97.6)	482.4 (105.2)
Within group	∆	9.7 (60.2) CI: −30.0 to 10.7	−11.3 (50.9) CI: −19.4 to 42.1	21.5 (62.8) CI: −48.7 to 5.6
	*p*-value	0.341	0.439	0.114
Between group	∆		−32.9 (20.4) CI: −74.4 to 6.7	
	*p*-value		0.117	
HGS, kg	n	38	14	24
	Baseline	34.2 (11.9)	28.7 (10.9)	37.4 (11.5)
	6 weeks	33.6 (11.1)	31.8 (13.5)	34.7 (9.7)
Within group	∆	−0.6 (6.3) CI: −1.5 to 2.6	3.1 (5.7) CI: −6.4 to 0.1	−2.7 (5.6) CI: 0.4 to 5.1
	*p*-value	0.578	0.059	0.026 *
Between group	∆		5.9 (1.9) CI:2.0 to 9.7	
	*p*-value		0.004 **	
PA, No. steps	n	22	7	15
	Baseline	4549.9 (2759.8)	4865.9 (3141.2)	4402.5 (2668.8)
	6 weeks	4897.9 (2796.2)	5091.7 (2808.8)	4807.4 (2884.4)
Within group	∆	348 (2071.6) CI: 1266.5 to 570.6)	225.8 (2783.1) CI: 2799.7 to 2348.1	405.0 (1762.7) CI: −1381.1 to 571.2
	*p*-value	0.440	0.837	0.389
Between group	∆		179.2 (970.9) CI: −1846 to 2204.4	
	*p*-value		0.855	

* statistical significance <0.05; ** statistical significance <0.001; CSA = cross sectional area; SMI = skeletal muscle index; KPS = Karnofsky performance status; self PF = self-reported physical function; 6MWT = six-minute walk test; HGS = handgrip strength; PA = physical activity; ∆ = difference from baseline to week six.
